# Supplementary material for: 6-Methoxymellein Isolated from Carrot (Daucus carota L.) Targets Breast Cancer Stem Cells by Regulating NF-κB Signaling
Source: Molecules. 2020 Sep 23;25(19):4374. doi: 10.3390/molecules25194374 (PMC7583823; doi:10.3390/molecules25194374)
Supplement: Supplementary file 1 [file molecules-25-04374-s001.pdf]

Table S1. Specific Real-time RT-qPCR primer sequences containing *IL-6, IL-8, and β-actin* genes

Genes	Primers
IL-6	Forward: 5’-AGACAGCCACTCACCTCTTCAG-3’<br>Reverse: 5’-TTCTGCCAGTGCCTCTTTGCTG-3’
IL-8	Forward : 5′-ATGACTTCCAAGCTGGCCGT-3′,<br>Reverse : 5′-TCCTTGGCAAAACTGCACCT-3′
β-actin	Forward: 5’-TGTTACCAACTGGGACGACA-3’<br>Reverse : 5’-GGGGTGTTGAAGGTCTCAAA-3

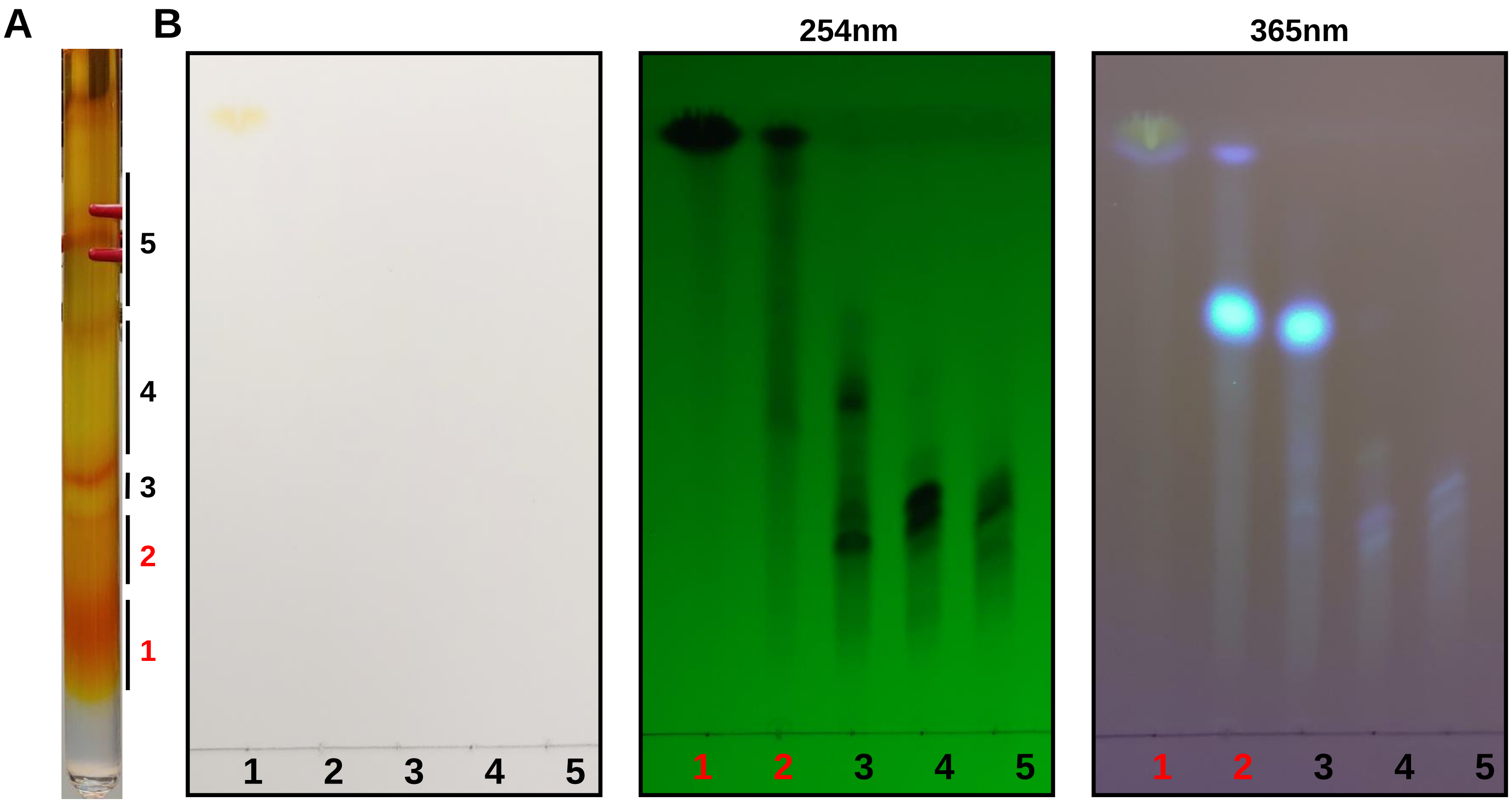

Figure S1. The purification procedure of a mammosphere formation inhibitor derived from carrot extracts using silica gel column chromatography. (A) The sample was isolated by silica gel chromatography with a solvent mixture [$CHCl_3$: MeOH (10:1)]. (B) TLC plate analysis of the purified sample ($CHCl_3$: MeOH = 10:1). Active fraction: #1 and #2.

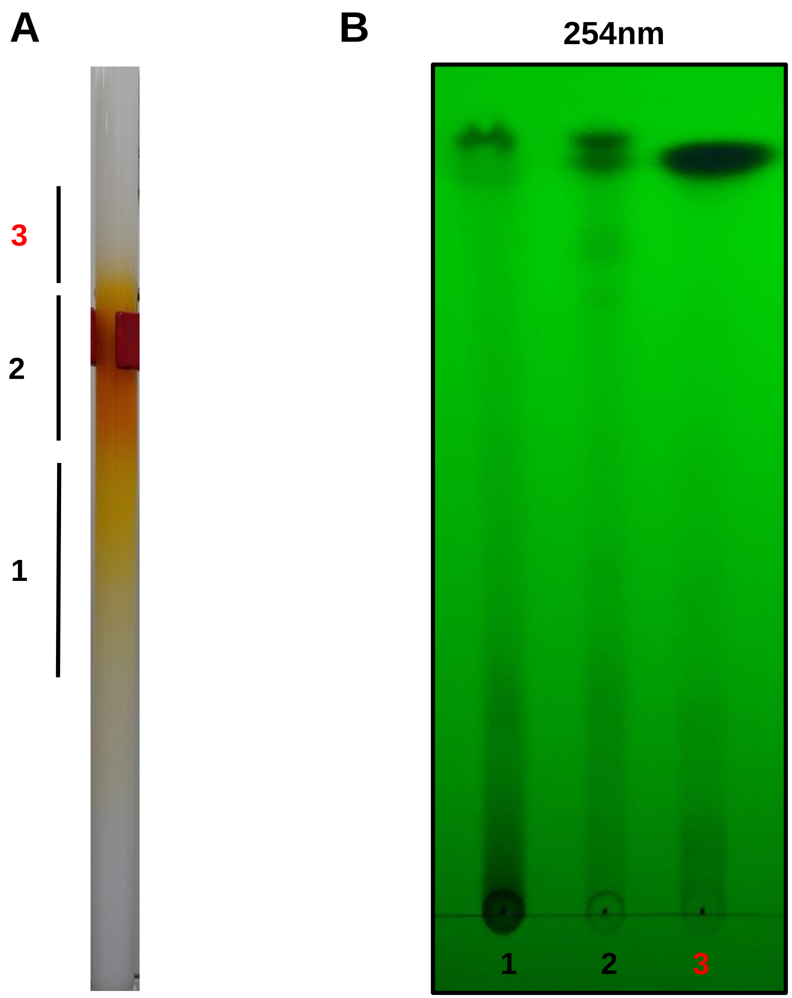

Figure S2. The purification procedure of a mammosphere formation inhibitor derived from carrot using Sephadex LH-20 column chromatography. (A) The sample was isolated by Sephadex LH-20 chromatography with MeOH. (B) TLC plate analysis of the purified sample ($CHCl_3$: MeOH = 10:1). Active fraction: #3.

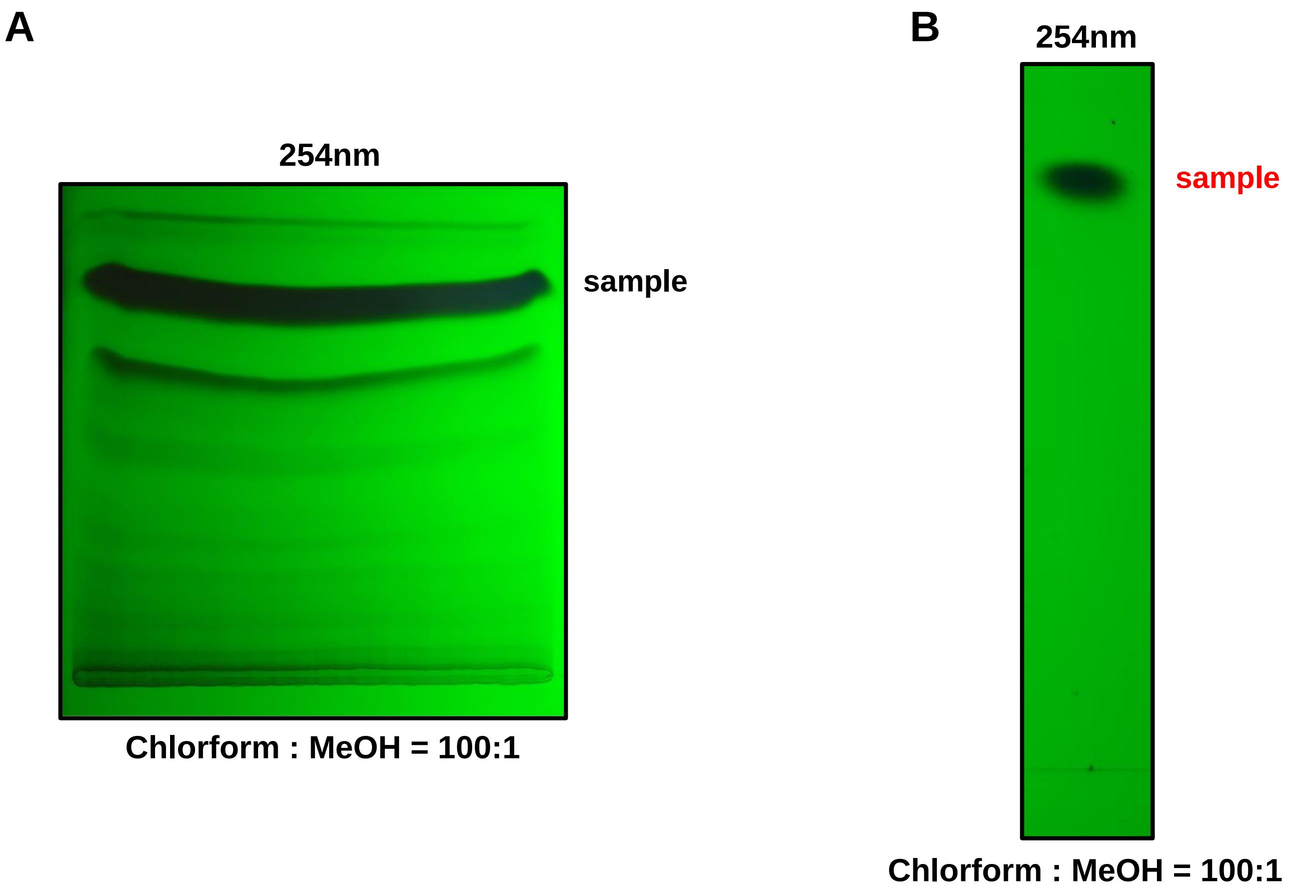

Figure S3. The purification procedure of a mammosphere formation inhibitor derived from carrot using preparative thin layer chromatography with $CHCl_3$:MeOH (100:1). (A) Preparatory TLC chromatography. (B) TLC plate analysis of the prepared TLC band after the samples were scraped and purified ($CHCl_3$: MeOH = 100:1).

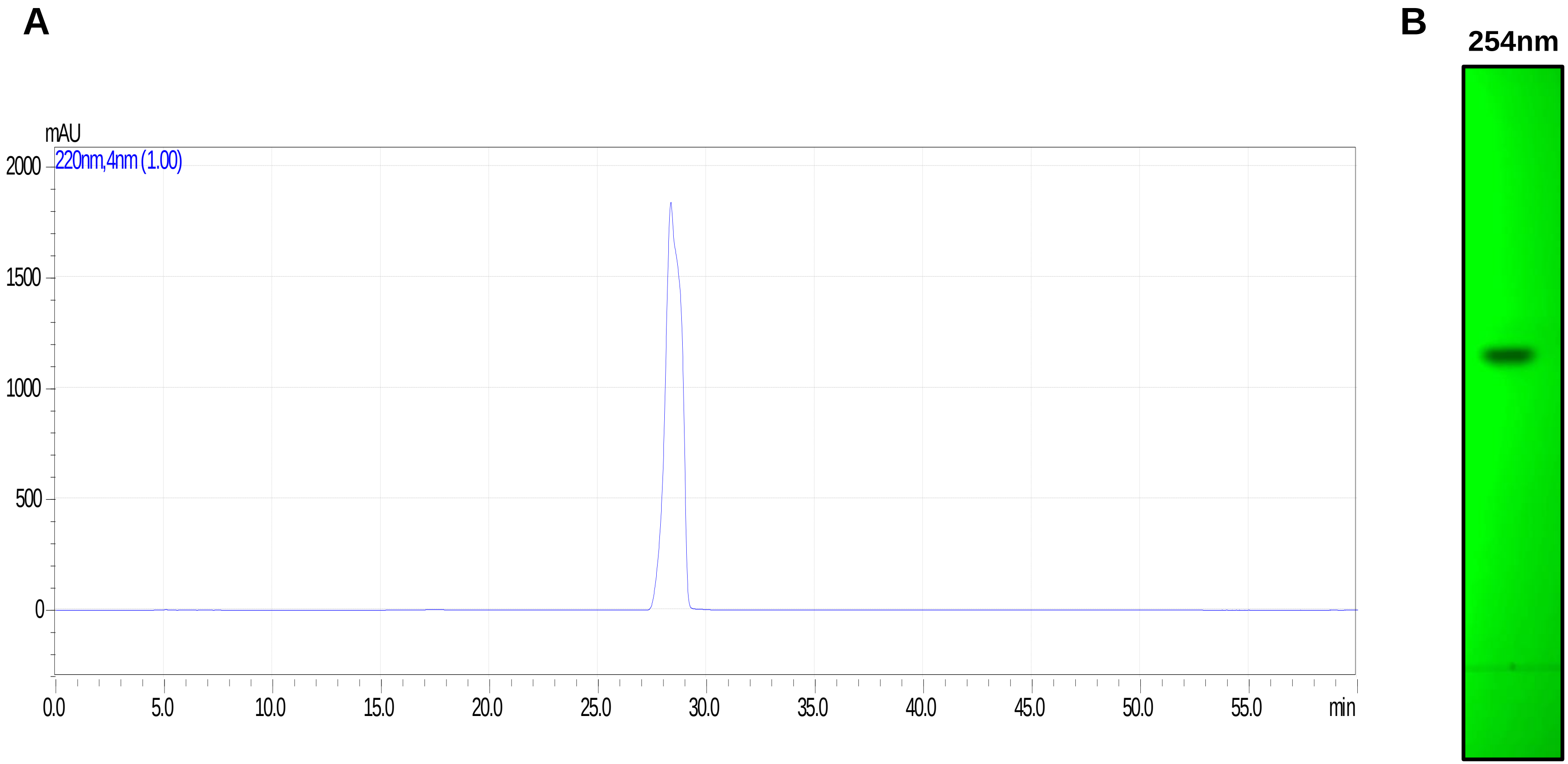

Figure S4. Purification procedure of a mammosphere formation inhibitor derived from carrot using HPLC. (A) Assessment of the major fractions using HPLC at one wavelength. Samples were collected based on the 220 nm wavelength. (B) TLC plate analysis of the purified sample ($CHCl_3$: MeOH = 100:1).

H-NMR

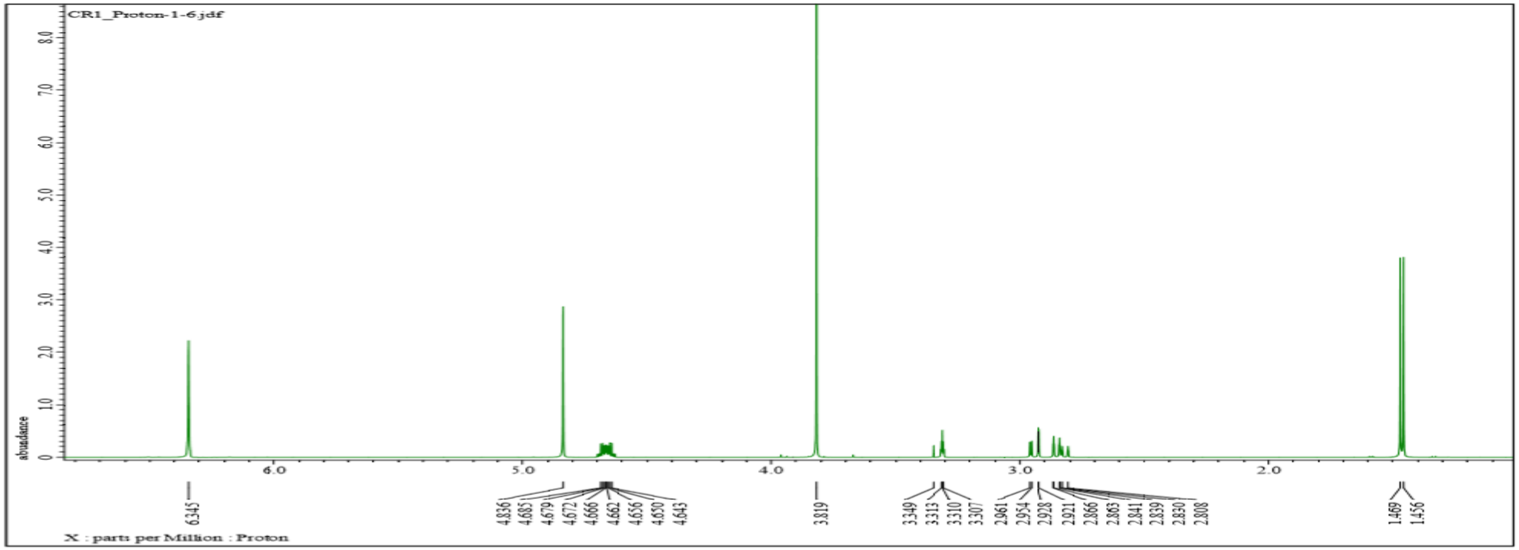

C-NMR

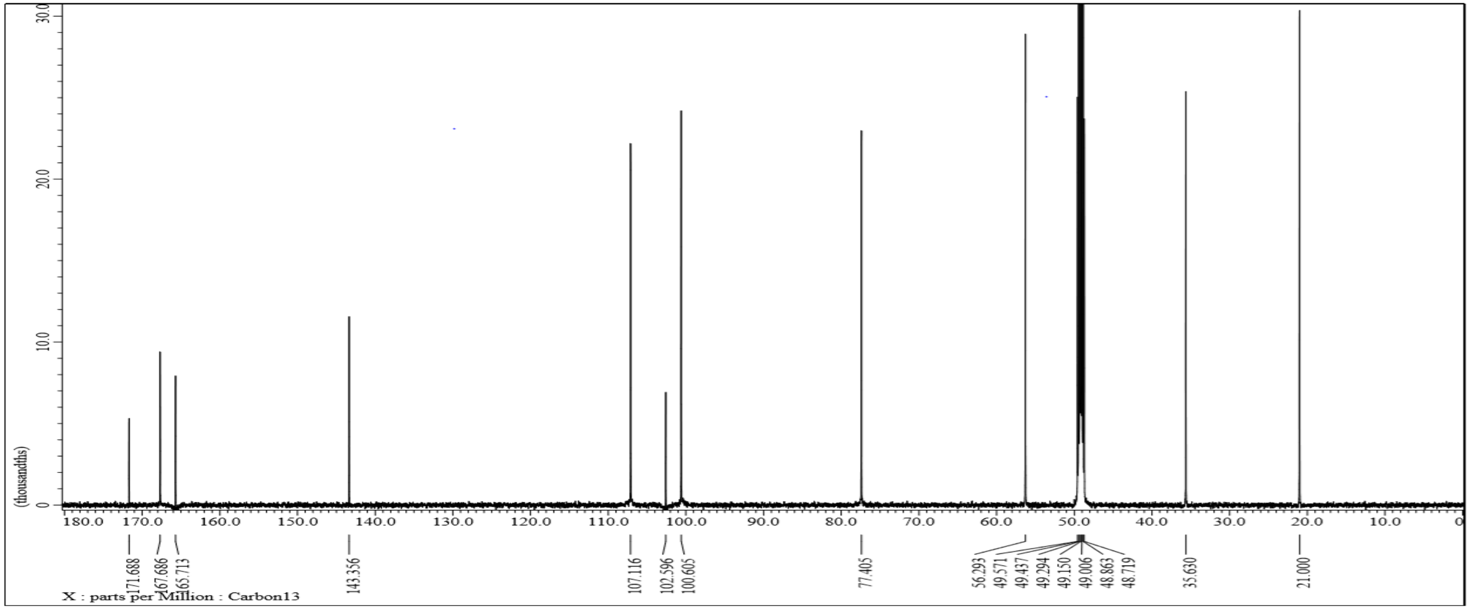

Figure S5. ^{1}H and ^{13}C NMR spectra of the purified sample.

HMQC

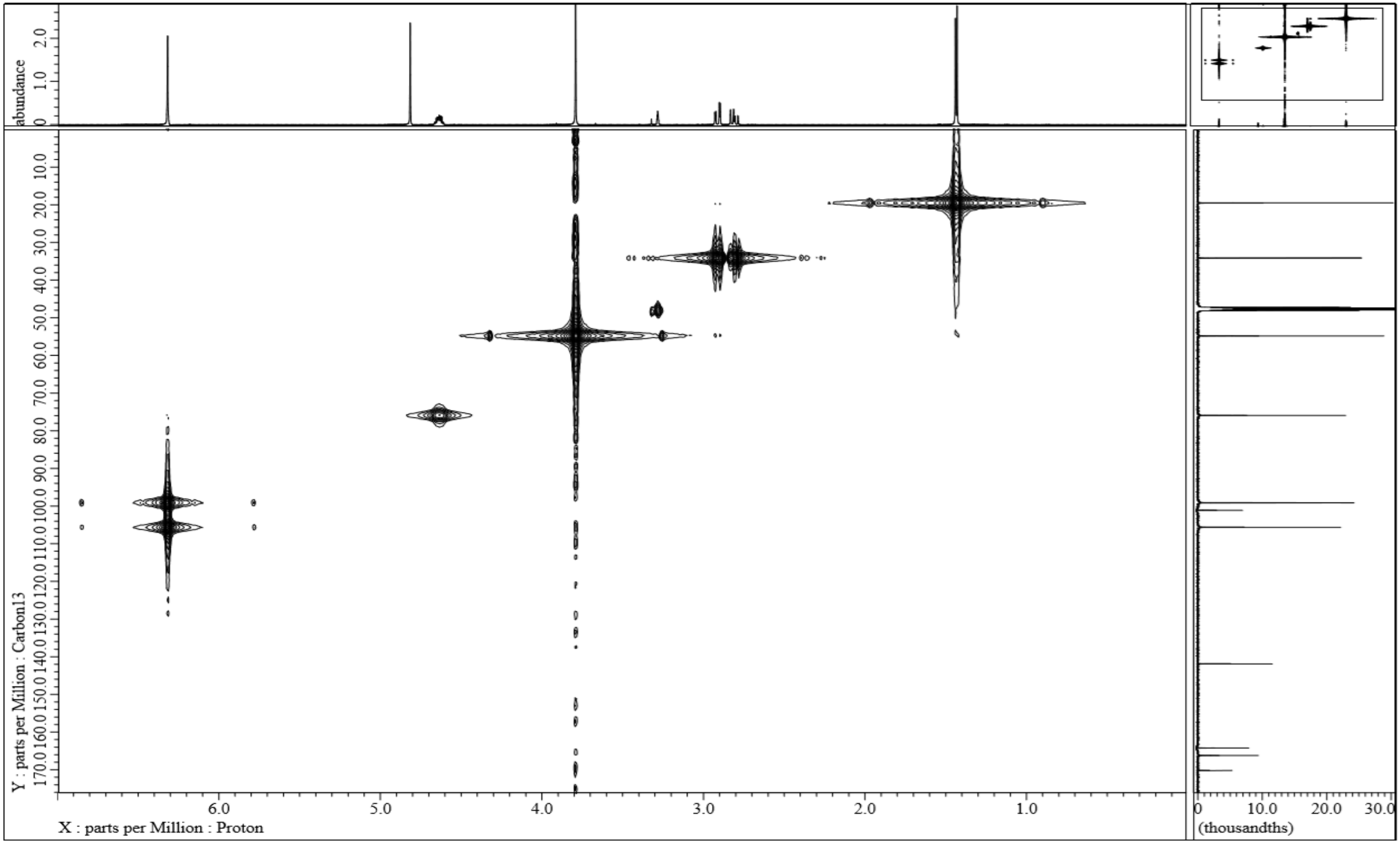

Figure S6. HMQC spectrum of the purified sample.

COSY

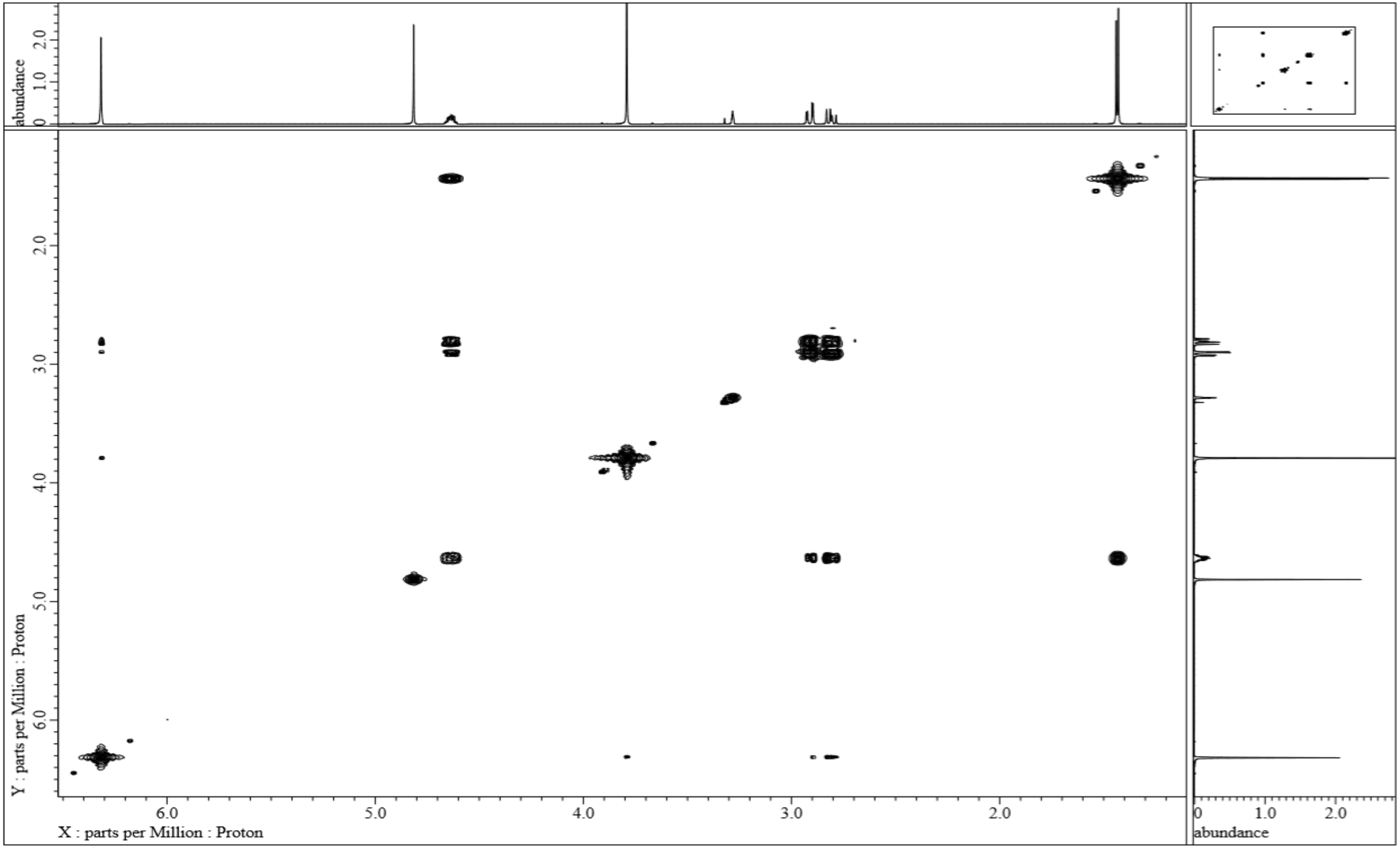

Figure S7. ^{1}H -^{1}H COSY spectrum of the purified sample.

HMBC

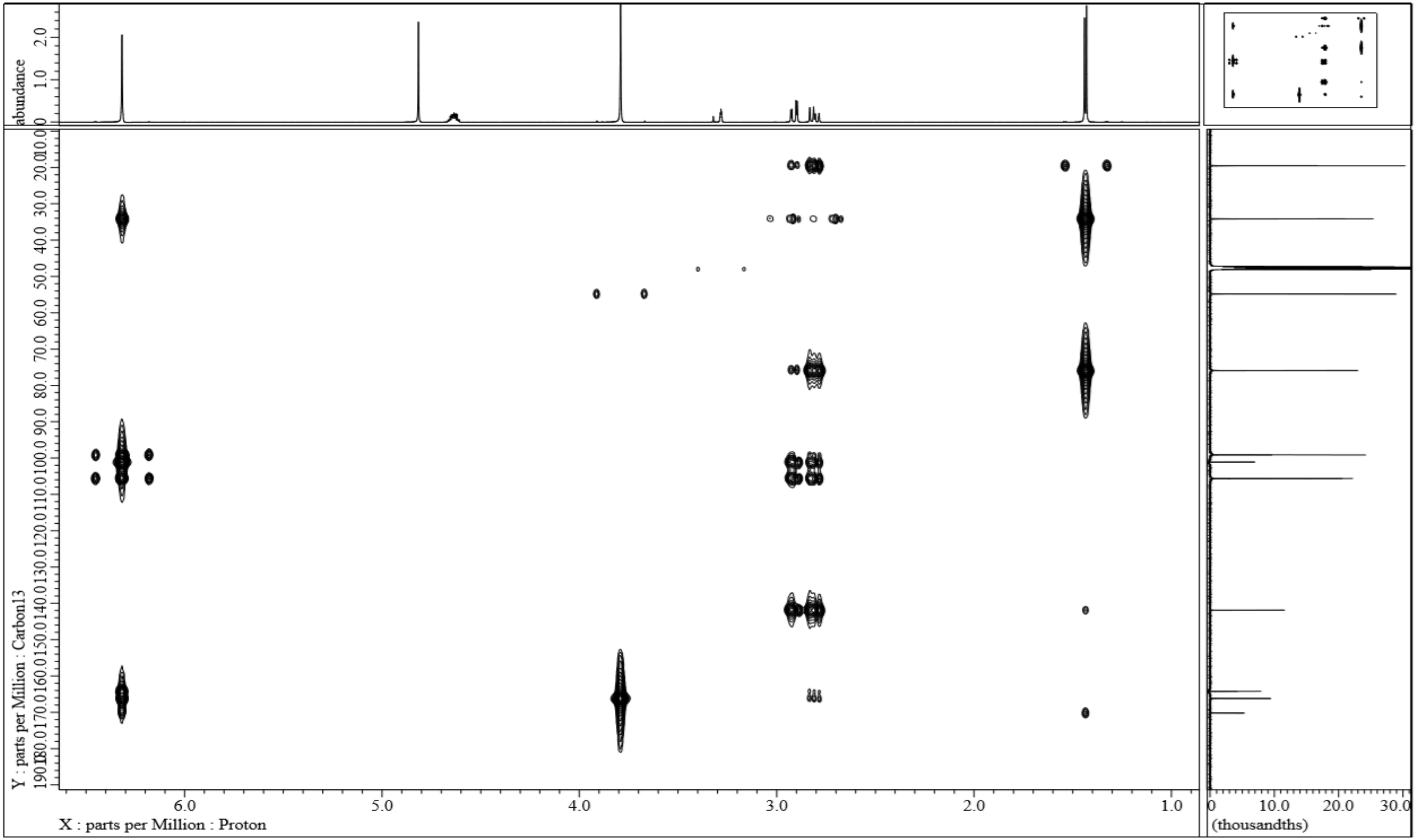

Figure S8. HMBC spectrum of the purified sample.

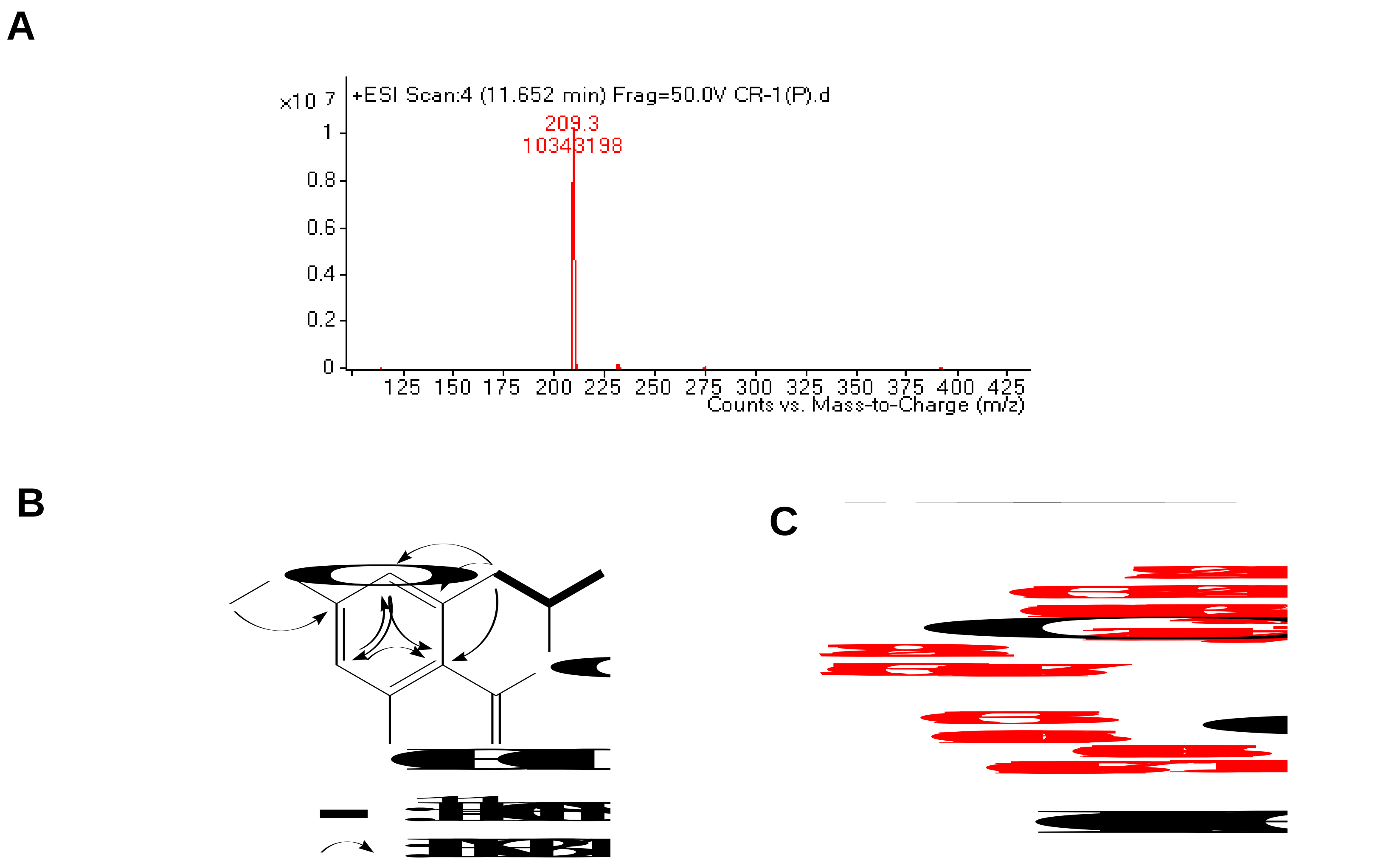

Figure S9. ESI mass spectrometry (A), two-dimensional NMR data (B), and ^{1}H and ^{13}C peak assignments (C) of the purified sample.
